# Expression of CYP1A1, CYP1B1 and MnSOD in a panel of human cancer cell lines

**DOI:** 10.1007/s11010-013-1758-8

**Published:** 2013-07-20

**Authors:** Hanna Piotrowska, Malgorzata Kucinska, Marek Murias

**Affiliations:** Department of Toxicology, Poznan University of Medical Sciences, ul. Dojazd 30, 60-631 Poznan, Poland

**Keywords:** CYP1A1, CYP1B1, Mitochondrial superoxide dismutase, Human cancer cell line

## Abstract

The expression of P450 enzymes and antioxidative enzymes in tumour tissue can have a major impact on the responsiveness of tumours to cancer chemotherapeutic drugs, therefore such information may be very precious when experiments are designed. The compressive information, concerning the expression of drug metabolism enzymes or antioxidative enzymes is still lacking, therefore in this study the expression of CYP1A1, CYP1B1 and mitochondrial superoxide dismutase MnSOD (both mRNA and protein) in a panel of eight commonly used cancer cell lines, representing four tumour tissues was assayed. In the study two ovarian cancer cell lines A2780 and SKOV-3, two colorectal cancer LOVO and DLD-1, two breast cancer derived MCF-7 and MDA-MB-231 and two cervical cancer cell lines HeLa and C33A were employed. The relatively high expression of all assayed enzymes was shown in MDA-MB-231 breast cancer cells, lack of cancer cell specific CYP1B1 protein was discovered in LOVO colorectal cells. In order to test possible correlation between expression of CYP1A1, CYP1B1 and MnSOD and modulators of their activity, cytotoxicity of resveratrol and its promising hydroxylated analogue 3,3′,4,4′,5,5′-*trans*-hexahydroxystilbene against cell lines used in experiment was assayed. The relatively high correlation was found between IC_50_ values calculated for 3,3′,4,4′,5,5′-*trans*-hexahydroxystilbene and expression of MnSOD (*r* = 0.6562).

**Electronic supplementary material:**

The online version of this article (doi:10.1007/s11010-013-1758-8) contains supplementary material, which is available to authorized users.

## Introduction

A cancer cell culture has been used as a valuable tool for discovery and development of new anticancer drugs for decades. They are used for academic, clinical and industrial research. Although, the most commonly used cell lines were applied in experiments described in thousands of papers, even the most important cell culture collections do not provide compressive information describing their biological properties like, e.g. the expression of drug metabolizing enzymes, drug transporters, receptors or antioxidative enzymes. Only few experimental papers describing and comparing the expression and/or activity of crucial for cancer cell enzymes and factors have been published so far, e.g. in a panel of cancer cell lines activity of P450 enzyme was described by Yu et al. [1], microRNA expression profiles were presented by Blower et al. [2], global microRNA analysis was performed by Solkilde et al. [3], while expression of nuclear receptors was described by Holbeck et al. [4]. Compresive analysis of p53 status in cancer cell lines was provided by Berglind et al. [5] using UMD_p53 database available on http://p53.free.fr.

Since the expression of P450 enzymes and antioxidative enzymes in tumour tissue can have a major impact on the responsiveness of tumours to cancer chemotherapeutic drugs, such information may be very precious when experiments are designed. Information regarding expression of key drug metabolizing enzymes and antioxidative enzymes may be also useful when prodrugs activated by cellular metabolic systems are prepared [6–9].

P450 cytochromes are enzymes which catalyse Phase-I metabolism reactions. These family haem-containing enzymes catalyse C-, N- and S-oxidation and dealkylation reactions of both xenobiotics and endobiotics. P450 1A1 (CYP1A1) is, from a pharmacological point of view, one of the most important members of the CYP family. CYP1A1 participates in the metabolism of a large number of xenobiotics, as well as a small number of endogenous substrates. CYP1A1 is responsible for the metabolism of various drugs, food components, and environmental contaminants. At the same time hydroxylation at a vacant position of an aromatic ring belongs to the most important reactions catalysed by this enzyme. This reaction is believed to be a critical step for the initiation of carcinogenesis, through the formation of highly reactive conversion products that can cause oncogenic and teratogenic mutations in experimental animals and humans [10, 11].

Another interesting member of the CYP1 subfamily is P450 1B1 (CYP1B1) cytochrome which is, similarly to CYP1A1, involved in the metabolism of xenobiotics and endobiotics. Similarly to CYP1A1, CYP1B1 activates several environmental mutagens, e.g. polycyclic aromatic hydrocarbons (PAHs), heterocyclic amines and aromatic amines [6]. CYP1B1 also catalyses the 4-hydroxylation of estrogens considered to be an important step in hormonal carcinogenesis [6]. Human CYP1B1 protein was detected in a variety of tumours but could not be detected in adjacent to normal tissues, where only mRNA was detected. This suggests that CYP1B1 could activate anticancer agents specifically in the cancer cells. The range of therapeutic strategies including CYP1B1-activated prodrugs as well as CYP1B1 inhibitors are currently tested [8].

The redox cycling of polyhydroxylated compounds catalysed by CYP1A1 and CYP1B1 may result in generation of superoxide radicals. Superoxide radical (O_2_^·^^−^) plays a central role in oxidative stress and impacts on the production of a plethora of other reactive oxygen species. The cellular and extracellular level of O_2_^·^^−^ is therefore controlled by the family of very efficient enzymes belonging to the superoxide dismutase (SOD) family. Cu, ZnSOD (SOD1) is located in cytosol, MnSOD (SOD2) is located in the mitochondrial matrix, while SOD3 is located in extracellular space [12, 13]. These enzymes catalyse the dismutation (disproportionation) of O_2_^·^^−^ to hydrogen peroxide and molecular oxygen and are essential to protect aerobic life from the toxic effects of oxygen [12, 13]. Some studies reported that MnSOD expression is elevated in cancer cells compared to normal tissue, including gastric and oesophageal [14, 15], colorectal [16], prostate [17] and lung cancer [18]. Moreover, MnSOD was shown in several reports to exert significant effect on growth and survival of cancer cells, for instance, changes in MnSOD levels in the cell affect the transcriptional activity of activator protein-1 (AP-1), dramatically increasing cells proliferation [19, 20]. It was reported by some authors that MnSOD overexpression may suprese tumour growth [21]; on the other hand, significant association between increased MnSOD activity and poor prognosis in cancer can be attributed to alterations in cancer cell migratory and invasive capacity [22]. Some reports describe MnSOD-p53 interactions [23–30]. Our own results suggest that ROS-generating agents may cause p53-mediated MnSOD downregulation and lead to induction of p53-transcriptional functions, which subsequently lead to the activation of mitochondrial driven apoptotic processes. MnSOD is therefore believed a key enzyme involved in the establishment of the cellular redox environment and controlling the biological status of cells.

Inhibition of CYP1 isoenzymes by resveratrol and methoxy- hydroxy- as well as thiomethylstilbenes was shown in several in vitro models [31–37]. This project is kind of pre-study preceding broader project aiming to evaluate relationship between CYP1A1, CYP1B1 and MnSOD and their effect on cytotoxic activity of resveratrol and its higher hydroxylated analogues. Therefore, as a first step we needed to screen expression of these enzymes in a panel of cancer cells and find possible correlation between their expression and cytotoxicity.

## Materials and methods

### Chemicals

Resveratrol analogue 3,3′,4,4′,5,5′-*trans*-hexahydroxystilbene (M12), was synthesized as described previously [38]. The RQ-PCR probes were provided by Roche (Mannheim, Germany). Probe numbers: 59, 61, 27, 60 and 23 were used for CYP1A1, CYP1B1, MnSOD, GADPH, and MRPL19 detection, respectively. The monoclonal antibodies were purchased from Santa Cruz Biotechnology (Dallas, TX USA). All other reagents used in experiments (including resveratrol) were purchased from Sigma-Aldrich (St. Louis, MO, USA).

### Cell culture

The cancer cell lines used in the experiment were purchased from the European Type Culture Collection (Sigma-Aldrich Co., St. Louis, MO, USA). The cell lines are listed in Table 1. The cells were maintained in phenol red-free DMEM medium supplemented with 10 % foetal bovine serum (FBS), 2 mM glutamine, penicillin (100 U/ml), and streptomycin (0.1 mg/ml). The cells were cultivated under standard conditions at 37 °C in a humidified atmosphere containing 5 % CO_2_ and 95 % air. For RNA isolation and western blot analysis the cells were seeded in a 6-well plates at a density of 1 × 10^6^ cells per well. All the cell culture chemicals were obtained from Sigma-Aldrich Co. St. Louis, MO, while all the cell culture consumables were provided by BD Falcon.

**Table 1:** Cancer cell lines used in experiment

Cell line	Tissue/karyotype (ETCC/ATCC description)	ETCC/ATCC description	Morphology/Growth mode	p53 status
DLD-1	Human colon adenocarcinoma/2*n* = 46, pseudodiploid	Derived from human colorectal adenocarcinoma. The cells have been used in the study of polar solvents on cell characteristics	Epithelial/adherent	Mutated [49, 50]
LOVO	Human colon adenocarcinoma/modal no. 49, (2*n* = 46)	Derived from a metastatic tumour in the left supraclavicular region of a 56-year-old male with adenocarcinoma of the colon. The cells produce carcino embryonic antigen (CEA)	Epithelial/adherent	Wt [49]
A2780	Human ovarian carcinoma/not specified	The A2780 human ovarian cancer cell line was established from tumour tissue from an untreated patient. Cells grow as a monolayer and in suspension in spinner cultures	Epithelial/adherent	Wt [51]
SKOV3	Human caucasian ovary adenocarcinoma/hypodiploid to hypotetraploid	Derived from the ascitic fluid from a 64-year-old Caucasian female with an ovarian tumour. Forms moderately well-differentiated adenocarcinoma consistent with ovarian primary cells	Epithelial/adherent	Wt [51]
MCF-7	Human Caucasian breast adenocarcinoma/2*n* = 46, hypertriploid to hypotetraploid	Established from the pleural effusion from a 69-year-old Caucasian female suffering from a breast adenocarcinoma. Cells exhibit some features of differentiated mammary epithelium including oestradiol synthesis and formation of domes. Cells may carry B or C type retrovirus and are considered to represent a category 2 pathogen (P2 containment). Cells express both the wild type and variant oestrogen receptors as well as progesterone receptor	Epithelial-like/adherent	Wt [52]
MDA-MB-231	Human caucasian adenocarcinoma/modal no.’s 62 and 64, near triploid	Isolated from pleural effusions of a breast cancer patient	Epithelial/adherent	Mutated [53]
HeLa	Human cervix epitheloid carcinoma/modal no.’s 62 and 64, near triploid	Derived from a cervical carcinoma from a 31-year-old female. This was the first aneuploid line derived from human tissue maintained in continuous cell culture. Susceptible to Poliovirus type I and adenovirus type 3. Identified as a contaminant in many other cell lines. The cells should be handled under laboratory containment level 2. Ethnicity: Black	Epithelial/adherent	Wt [54]
C-33A	Human Caucasian cervical carcinoma/hypodiploid	Derived from a cervical carcinoma from a 66-year-old female	Epithelial/Adherent	Mutated [55] 273/CGT>TGT

### Real-time quantitative PCR (RTq-PCR) analysis

Total RNA was isolated according to the method of Chomczynski and Sacchi [39].The RNA concentration was quantified by measuring the optical density (OD) at 260 nm and their integrity was confirmed by denaturing agarose gel electrophoresis. RNA samples were treated with DNAse I and reverse-transcribed into cDNA using oligo-dT primers. Reverse transcription was performed using M-MLV Reverse Transcriptase (Invitrogen, Carlsbad, CA, USA) according to manufacturer instructions.

RQ-PCR was conducted in the Light Cycler real-time PCR detection system Roche Diagnostics GmbH, (Mannheim, Germany) using a LightCycler^®^ 480 Probes Master kit. Target cDNA was quantified using the relative quantification method. The quantity of CYP1A1, CYP1B1 and MnSOD in each sample was standardized by GAPDH and MRPL19 (Table 2). For RTq-PCR analysis of CYP1A1, CYP1B1 and MnSOD mRNA expression, 1 μl of total (20 μl) cDNA solution was added to the mixture of the LightCycler^®^ 480 Probes Master kit (Roche, Mannheim, Germany), primers and probes for CYP1A1, CYP1B1 and MnSOD respectively. In case of negative control, cDNA was not added.

**Table 2:** Oligonucleotide sequences used for RTq-PCR analysis

Transcript	Sequence (5′-3′ direction)	Probe number	Gene accession number	Product size (bp)
CYP1A1	5′ ggggcgttgtgtctttgtaa 3′	59	NM_000499.3	64
	5′ tgggttgacccatagcttct 3′			
CYP1B1	5′ ggcattagagtcaactacacaaagc 3′	61	NM_000104.3	67
	5′ gaatggcaagtgccaaaaa 3′			
SOD-2	5′ gcactagcagcatgttgagc 3′	27	NM_001024466.1	76
	5′ gagcccagataccccaaaac 3′			
GAPDH	5′ ctctgctcctcctgttcgac 3′	60	NM_002046.3	112
	5′ acgaccaaatccgttgactc 3′			
MRPL19	5′ caattacacgcgtgaaccac 3′	23	NM_014763.3	62
	5′ ggtggagtaggcacattgaaa 3′			

### SDS-PAGE and western blot analysis

The cells were grown in 6 well plates. The cells were collected when they were 60 % confluent and dissolved in a RIPA buffer with proteinase inhibitors (30 min on ice) and centrifuged. The obtained supernatant was used for electrophoresis, protein concentration in supernatant was determined using Qubit fluorometer (Invitrogen) and Quant-iTTM Protein Assay Kit (Invitrogen, Burlington, Ontario,Canada). For electrophoresis, 30 μg of protein were resuspended in sample buffer and separated on 10 % Tris–glycine gel using SDS-PAGE. Gel proteins were transferred to nitrocellulose, which was blocked with 5 % milk in Tris buffered saline/Tween. Immunodetection was performed with rabbit polyclonal anti-CYP1A1 Ab (sc-20772), rabbit polyclonal anti-CYP1B1 Ab (sc-32882), anti-MnSOD Ab (sc-30080) followed by incubation with goat anti-rabbit HRP- conjugated Ab (sc-2004). The membranes were also incubated with anti-actin HRP conjugated Ab (sc-1616) to ensure equal protein loading of the lanes. Bands were revealed using SuperSignal West Femto maximum sensitivity substrate Pierce Biotechnology Inc. (Rockford, IL, USA). Densitometric quantification of band intensity was measured using ImageJ 1.46 software (NIH, USA) and was normalised relatively to the band intensity of the β-actin loading control.

### Cytotoxicity study

As recent data showed the MTT-reducing activity can be increased by the polyphenolic antioxidant resveratrol without a corresponding increase in the number of living cells, therefore a protein determination assay was used for the cytotoxicity experiments adapted from Seibert et al. [40]. Briefly, the cells were detached using trypsin, counted using Casy-1 cell counter (Scharfe System, Reutlingen Germany), and seeded into 96 well plates at the density of 10,000 cells/100 μL/well. Cells were allowed to attach overnight and incubated for 24 h with resveratrol and 3,3′,4,4′,5,5′-*trans*-hexahydroxystilbene in concentrations ranging from 1.5 to 100 μM in six replicates. DMSO was used as a control, and the concentration in medium did not exceed 0.1 %.Two groups of six wells receiving 0.1 % DMSO served as a control. After incubation, the medium was aspirated and the cell layers were washed three times with phosphate buffered saline (PBS). Cells were lysed by incubation with 0.5 N NaOH for 45 min at 37 °C. Protein content was determined Bio-Rad Protein Assay Kit (Bio-Rad Hercules, USA) modified for microtiter plates. Bovine serum albumin (BSA) (1.5–24 μg/well) was used to determine the standard curve. Absorption at 750 nm was read with a microtiter plate photometer (ELX800, Bio-TEK). The protein content of culture wells was compared to the mean protein content of the control cultures (12-wells per plate) and expressed as percentage of control. Experiments were replicated at least 2 times with cultures of different passages. The IC_50_ values were determined from semilogarithmic plots of the mean concentration-effect relationships.

### Statistical analysis

One-way ANOVA followed by Dunnett’s multiple comparisons test was performed using GraphPad Prism version 6.02 for Windows, GraphPad Software, La Jolla California USA, www.graphpad.com.

## Results

**Fig. 1:**
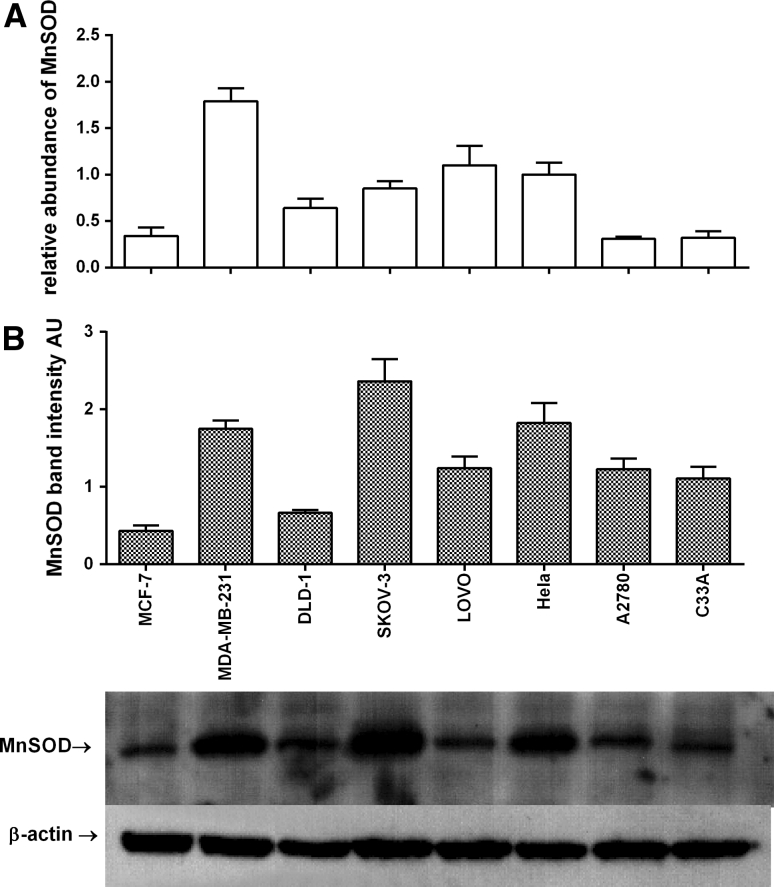
Gene and protein expression of MnSOD in tested cell lines. **A** RTq-PCR analyses; relative abundance of MnSOD mRNAs, **B** Western blot analyses of MnSOD bands, the bands of β-actin were measured to normalise the results. Densitometric quantification of the corresponding bands was performed using ImageJ 1.45 software. All results are presented and mean ± SD from three experiments. Detailed statistical analysis is provided in supplementary materials

**Fig. 2:**
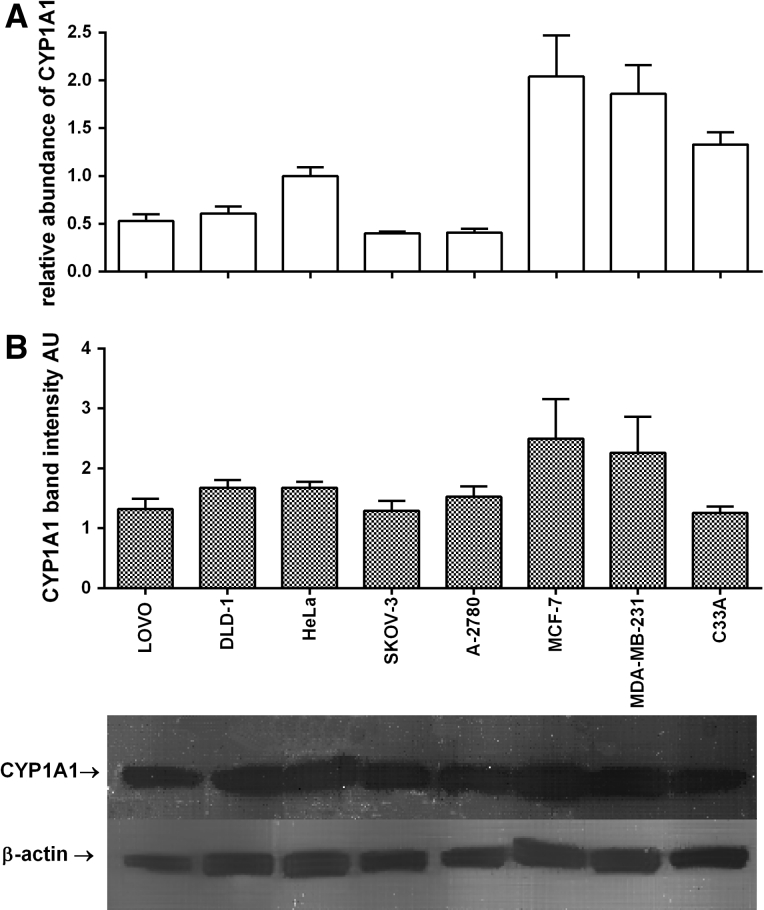
Gene and protein expression of CYP1A1 in tested cell lines. **A** RTq-PCR analyses; relative abundance of CYP1A1 mRNAs, **B** Western blot analyses of CYP1A1 bands, the bands of β-actin were measured to normalise the results. Densitometric quantification of the corresponding bands was performed using ImageJ 1.45 software. All results are presented and mean ± SD from three experiments. Detailed statistical analysis is provided in supplementary materials

**Fig. 3:**
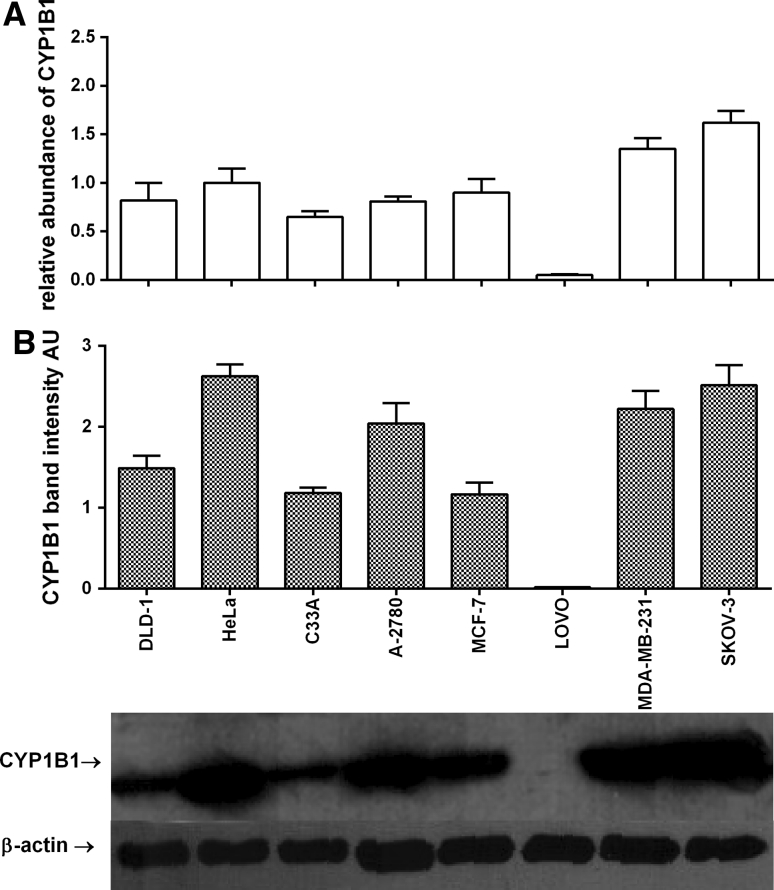
Gene and protein expression of CYP1B1 in tested cell lines. **A** RTq-PCR analyses; relative abundance of CYP1B1 mRNAs, **B** Western blot analyses of CYP1B1 bands, the bands of β-actin were measured to normalise the results. Densitometric quantification of the corresponding bands was performed using ImageJ 1.45 software. All results are presented and mean ± SD from three experiments. Detailed statistical analysis is provided in supplementary materials

In this study expression of cytochrome P450 (CYP) isoforms CYP1A1, CYP1B1 as well as MnSOD in 8 cell lines representing 4 tumuor tissues is presented. In the tested cells the highest MnSOD mRNA expression was found in MDA-MB-231 cells (RA = 1.79 ± 0.14), while the lowest expression was found in MCF-7 (RA = 0.34 ± 0.09), C33A (RA = 0.32 ± 0.07) and A2780 (RA = 0.31 ± 0.02) cell lines. These results were in agreement with MnSOD protein level measured using the western blot technique. The only exception was LOVO cell line. In this colorectal cell line the level of MnSOD protein was relatively high when compared with mRNA (Fig. 1). The highest level of CYP1A1 mRNA was found in both breast cancer cell lines: MCF-7 (RA = 2.04 ± 0.43) and MDA-MB-231(RA = 1.86 ± 0.30) used in our screening; similarly the highest level of CYP1A1 protein was found in these lines (Fig. 2). The most interesting results were obtained for CYP1B1; the highest level of CYP1B1 protein was again measured in HeLa and SKOV-3 cells followed by MDA-MB-231 cells. CYP1B1 mRNA and protein level in LOVO cells can be presented as the most interesting results obtained in our study. The CYP1B1 mRNA level was very low (RA = 0.05 ± 0.01) while CYP1B1 protein was not present in colorectal LOVO cancer cells (Fig. 3). Detailed statistical analysis is provided in supplementary materials (Tables 1s, 2s, 3s).

**Fig. 4:**
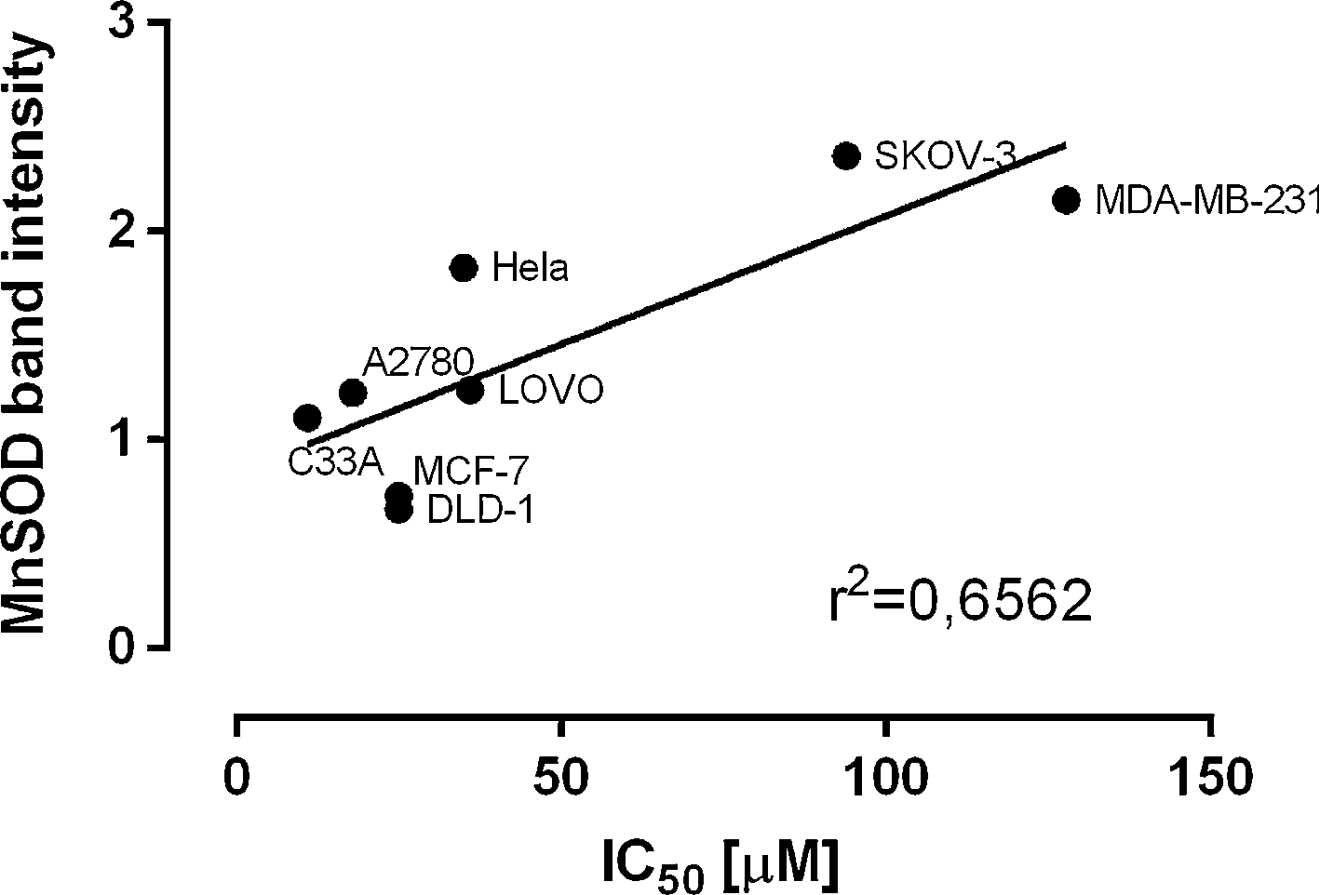
Plot of IC_50_ values obtained for 3,3′,4,4′,5,5′-*trans*-hexahydroxystilbene (M12) in cytotoxicity study versus Western blot bands intensities of MnSOD

In order to test possible relationship between the expression of CYP1A1, CYP1B1 and MnSOD and cytotoxicity of compounds tested in our laboratory we were able to analyse the relationship between cytotoxic activity of resveratrol (3,4′,5-*trans*-hydroxystilbene) and 3,3′,4,4′,5,5′-*trans*-hexahydroxystilbene (M12). The IC_50_ values obtained in cytotoxicity studies are presented in Table 3. The relatively high correlation (*r* ^2^ = 0.6562) between expression of MnSOD and cytotoxicity of M12 was found and presented in Fig. 4, all other relationships are presented in Fig. 1s.

**Table 3:** Cytotoxic activity of resveratrol and M12 against cell lines used in experiment

Cell line	Resveratrol IC_50_ (μM)	M12 IC_50_ (μM)
MCF-7	49.7 ± 9.4	25.6 ± 6.1
MDA-MB-231	38.1 ± 5.4	127.8 ± 1.6
DLD-1	42.7 ± 1.1	25.3 ± 2.6
SKOV-3	44.4 ± 9.8	94.4 ± 1.5
LOVO	57.0 ± 8.4	36.7 ± 4.0
Hela	53.9 ± 1.3	35.2 ± 5.8
A2780	35.4 ± 2.5	18.4 ± 0.9
C33A	72.5 ± 4.7	11.6 ± 2.5

## Discussion

Cancer cell lines are extensively used for various experiments in scientific laboratories worldwide. Although, a plethora of data is generated every day in the field of cancer cells research, there is a need for systematization and collation of available information about their properties like expression of enzymes, receptors or transcription factors. The expression of drug metabolizing and antioxidative enzymes is a crucial parameter for designing of anticancer drugs [41, 42]. Differences in the expression of drug metabolizing enzymes and drug transporters in cancer cells, were shown in several reports and were used to explain diverse effect of several natural products and anticancer agents against different cancer cell lines. For instance, it was shown that the expression of organic anion-transporting polypeptides 1B1 and 1B3 in ovarian cancer OVCAR-3 and SKOV-3 cells may modulate paclitaxel disposition during therapy [43], while the expression of sulfotransferase 1A1 may modify growth of breast cancer cells incubated with resveratrol [44]. The activation of the natural product eupatorin, which is attributed to CYP1A1 expression in MDA-MB-486 cells, but not normal MCF-10A cells, was described by Androutsopoulos and coworkers [45]. It was also suggested that different CYP1B1 expression patterns in ovarian cell lines A2780 and SKOV-3 may affect their sensitivity to cytotoxic activity of 3,4,4′,5-tetramethoxystilbene (DMU-212) [46]. In our opinion data presented in this paper may be very helpful in designing similar experiments. On the other hand, this information may be also useful in designing experiments employing transfection of these cells with siRNA or cDNA targeting CYP1A1, CYP1B1 and MnSOD. For example, such experiments were used for explanation of activation of 2-amino-1-methyl-6-phenylimidazo[4,5-b]pyridine by CYP1A1 on mutagenesis and DNA damage in CHO cells [47], while experiments employing CYP1B1 transfected cells were used to explain its role in activation of docetaxel [48]. As it was shown in our cytotoxicity study, the correlations between the P450 or MnSOD activities and the patterns of toxicity of anticancer agents may also aid in the design of further experiments to evaluate new hypotheses regarding the role of P450 and MnSOD enzymes in the metabolism of selected anticancer agents. The most interesting results of our study showed, that level of MnSOD expression in cancer cells may modulate cytotoxic effect exerted by superoxide generating compounds like, e.g. M12, this hypothesis, however, should be further evaluated using cells stably transfected with MnSOD.

## Electronic supplementary material

Below is the link to the electronic supplementary material.

Supplementary material 1 (DOCX 369 kb)

## Abbreviations

A2780: Human ovarian carcinoma cell line
AP-1: Activator protein 1
ATCC: American Type Culture Collection
C33A: Human cervical carcinoma
CYP: Cytochrome P450
DLD-1: Human colon adenocarcinoma cell line
DMEM: Dulbecco’s modified Eagle medium
ECACC: European Collection of Cell Cultures
FBS: Foetal bovine seum
GAPDH: Glyceraldehyde 3-phosphate dehydrogenase
HeLa: Human cervix epitheloid carcinoma cell line
HRP: Horseradish peroxidase
LoVo: Human colon adenocarcinoma cell line
M-MVL: Moloney murine leukaemia virus
M12: 3,3′,4,4′,5,5′-*trans*-Hexahydroxystilbene
MCF-7: Human Caucasian breast adenocarcinoma
MDA-MB-231: Human Caucasian breast adenocarcinoma
MnSOD: Mitochondrial superoxide dismutase
MRPL19: Mitochondrial ribosomal protein L19
OD: Optical density
OVCAR-3: Human ovarian carcinoma cell line
PAHs: Polycyclic aromatic hydrocarbons
RA: Relative abundance
RQ-PCR: Real time quantitative PCR
SDS: Sodium dodecyl sulphate
SKOV-3: Human Caucasian adenocarcinoma cell line
CuZnSOD (SOD1): Cytosolic superoxide dismutase
MnSOD (SOD2): Mitochondrial superoxide dismutase
SOD3: Extracellular superoxide dismutase
