# Supplementary material for: Expression of CYP1A1, CYP1B1 and MnSOD in a panel of human cancer cell lines
Source: Mol Cell Biochem. 2013 Jul 20;383(1):95–102. doi: 10.1007/s11010-013-1758-8 (PMC3788183; doi:10.1007/s11010-013-1758-8)
Supplement: Supplementary file 1 — Supplementary material 1 (DOCX 369 kb) [file 11010_2013_1758_MOESM1_ESM.docx]

**SUPLEMENTARY INFORMATION**

Expression of CYP1A1, CYP1B1 and MnSOD in panel of human cancer cell lines.

Hanna Piotrowska^#^, Malgorzata Kucinska^#^, Marek Murias*

^#^ both authors equally contributed to this work

Department of Toxicology, Poznan University of Medical Sciences, Poznan Poland

*Correspondence:

Marek Murias,

Poznan University of Medical Sciences,

Department of Toxicology,

ul. Dojazd 30,

60-631 Poznań, Poland,

Phone +48 61 8472081, Fax +48 61 8470721;

e-mail: marek.murias@ump.edu.pl

**Table of contents**

| Tab.1s Differences in expression of of MnSOD (mRNA and protein) in cancer cell lines used in experiment. For comparisons one-way ANOVA multiple comparison test followed by Tukey’s test was used. | 3 |
| --- | --- |
| Tab.2s Differences in expression of of CYP1A1 (mRNA and protein) in cancer cell lines used in experiment. For comparisons one-way ANOVA multiple comparison test followed by Tukey’s test was used. | 4 |
| Tab.3s Differences in of CYP1B1 in cancer cell lines used in experiment (mRNA and protein). For comparisons one-way ANOVA multiple comparison test followed by Tukey’s test was used. | 5 |
| Ryc. 1s Plot of cytotoxic activity of resveratrol and its analogue 3,3’,4,4’,5,5’-*trans*-hexahydroxystilbene (M12) versus band intensities of MnSOD, CYP1A1 and CYP1B1. | 6 |

| **Tab 1s** |  | MnSOD mRNA (RTPCR) | |  |  |  | MnSOD protein (Western blot) | | |
| --- | --- | --- | --- | --- | --- | --- | --- | --- | --- |
| Tukey's multiple comparisons test | Mean Diff, | 95% CI of diff, | Significant? | Summary |  | Mean Diff, | 95% CI of diff, | Significant? | Summary |
| MCF-7 vs. MDA-MB-231 | -1,421 | -1,901 to -0,9403 | Yes | **** |  | -1,45 | -1,782 to -1,118 | Yes | **** |
| MCF-7 vs. DLD1 | 0,06439 | -0,4159 to 0,5447 | No | ns |  | -0,3 | -0,6321 to 0,03208 | No | ns |
| MCF-7 vs. SKOV-3 | -1,632 | -2,112 to -1,151 | Yes | **** |  | -0,51 | -0,8421 to -0,1779 | Yes | ** |
| MCF-7 vs. LOVO | -0,5087 | -0,9890 to -0,02838 | Yes | * |  | -0,76 | -1,092 to -0,4279 | Yes | **** |
| MCF-7 vs. HeLa | -1,095 | -1,575 to -0,6148 | Yes | **** |  | -0,66 | -0,9921 to -0,3279 | Yes | **** |
| MCF-7 vs. A2780 | -0,4958 | -0,9761 to -0,01546 | Yes | * |  | 0,03 | -0,3021 to 0,3621 | No | ns |
| MCF-7 vs. C33A | -0,3764 | -0,8567 to 0,1040 | No | ns |  | 0,02 | -0,3121 to 0,3521 | No | ns |
| MDA-MB-231 vs. DLD1 | 1,485 | 1,005 to 1,965 | Yes | **** |  | 1,15 | 0,8179 to 1,482 | Yes | **** |
| MDA-MB-231 vs. SKOV-3 | -0,2109 | -0,6913 to 0,2694 | No | ns |  | 0,94 | 0,6079 to 1,272 | Yes | **** |
| MDA-MB-231 vs. LOVO | 0,9119 | 0,4316 to 1,392 | Yes | *** |  | 0,69 | 0,3579 to 1,022 | Yes | **** |
| MDA-MB-231 vs. HeLa | 0,3255 | -0,1548 to 0,8059 | No | ns |  | 0,79 | 0,4579 to 1,122 | Yes | **** |
| MDA-MB-231 vs. A2780 | 0,9249 | 0,4445 to 1,405 | Yes | *** |  | 1,48 | 1,148 to 1,812 | Yes | **** |
| MDA-MB-231 vs. C33A | 1,044 | 0,5640 to 1,525 | Yes | **** |  | 1,47 | 1,138 to 1,802 | Yes | **** |
| DLD1 vs. SKOV-3 | -1,696 | -2,176 to -1,216 | Yes | **** |  | -0,21 | -0,5421 to 0,1221 | No | ns |
| DLD1 vs. LOVO | -0,5731 | -1,053 to -0,09277 | Yes | * |  | -0,46 | -0,7921 to -0,1279 | Yes | ** |
| DLD1 vs. HeLa | -1,159 | -1,640 to -0,6792 | Yes | **** |  | -0,36 | -0,6921 to -0,02792 | Yes | * |
| DLD1 vs. A2780 | -0,5602 | -1,041 to -0,07985 | Yes | * |  | 0,33 | -0,002078 to 0,6621 | No | ns |
| DLD1 vs. C33A | -0,4408 | -0,9211 to 0,03957 | No | ns |  | 0,32 | -0,01208 to 0,6521 | No | ns |
| SKOV-3 vs. LOVO | 1,123 | 0,6425 to 1,603 | Yes | **** |  | -0,25 | -0,5821 to 0,08208 | No | ns |
| SKOV-3 vs. HeLa | 0,5365 | 0,05615 to 1,017 | Yes | * |  | -0,15 | -0,4821 to 0,1821 | No | ns |
| SKOV-3 vs. A2780 | 1,136 | 0,6555 to 1,616 | Yes | **** |  | 0,54 | 0,2079 to 0,8721 | Yes | *** |
| SKOV-3 vs. C33A | 1,255 | 0,7749 to 1,736 | Yes | **** |  | 0,53 | 0,1979 to 0,8621 | Yes | *** |
| LOVO vs. HeLa | -0,5864 | -1,067 to -0,1061 | Yes | * |  | 0,1 | -0,2321 to 0,4321 | No | ns |
| LOVO vs. A2780 | 0,01292 | -0,4674 to 0,4933 | No | ns |  | 0,79 | 0,4579 to 1,122 | Yes | **** |
| LOVO vs. C33A | 0,1323 | -0,3480 to 0,6127 | No | ns |  | 0,78 | 0,4479 to 1,112 | Yes | **** |
| HeLa vs. A2780 | 0,5993 | 0,1190 to 1,080 | Yes | ** |  | 0,69 | 0,3579 to 1,022 | Yes | **** |
| HeLa vs. C33A | 0,7187 | 0,2384 to 1,199 | Yes | ** |  | 0,68 | 0,3479 to 1,012 | Yes | **** |
| A2780 vs. C33A | 0,1194 | -0,3609 to 0,5997 | No | ns |  | -0,01 | -0,3421 to 0,3221 | No | ns |

| **Tab. 2s** |  | CYP1A1 mRNA (RTPCR) | |  |  |  | CYP1A1 protein (Western blot) | | |
| --- | --- | --- | --- | --- | --- | --- | --- | --- | --- |
| Tukey's multiple comparisons test | Mean Diff, | 95% CI of diff, | Significant? | Summary |  | Mean Diff, | 95% CI of diff, | Significant? | Summary |
| LOVO vs. DLD-1 | -0,08 | -0,6526 to 0,4926 | No | ns |  | -0,436 | -0,7729 to -0,09916 | Yes | ** |
| LOVO vs. HeLa | -0,47 | -1,043 to 0,1026 | No | ns |  | -0,382 | -0,7189 to -0,04512 | Yes | * |
| LOVO vs. SKOV-3 | -0,8 | -1,373 to -0,2274 | Yes | ** |  | -0,1208 | -0,4577 to 0,2161 | No | ns |
| LOVO vs. A-2780 | 0,12 | -0,4526 to 0,6926 | No | ns |  | -0,229 | -0,5658 to 0,1079 | No | ns |
| LOVO vs. MCF-7 | -1,51 | -2,083 to -0,9374 | Yes | **** |  | -0,2898 | -0,6267 to 0,04704 | No | ns |
| LOVO vs. MDA-MB-231 | -1,33 | -1,903 to -0,7574 | Yes | **** |  | -0,1289 | -0,4657 to 0,2080 | No | ns |
| LOVO vs. C33A | -0,8 | -1,373 to -0,2274 | Yes | ** |  | -0,03064 | -0,3675 to 0,3062 | No | ns |
| DLD-1 vs. HeLa | -0,39 | -0,9626 to 0,1826 | No | ns |  | 0,05404 | -0,2828 to 0,3909 | No | ns |
| DLD-1 vs. SKOV-3 | -0,72 | -1,293 to -0,1474 | Yes | ** |  | 0,3152 | -0,02162 to 0,6521 | No | ns |
| DLD-1 vs. A-2780 | 0,2 | -0,3726 to 0,7726 | No | ns |  | 0,2071 | -0,1298 to 0,5439 | No | ns |
| DLD-1 vs. MCF-7 | -1,43 | -2,003 to -0,8574 | Yes | **** |  | 0,1462 | -0,1907 to 0,4831 | No | ns |
| DLD-1 vs. MDA-MB-231 | -1,25 | -1,823 to -0,6774 | Yes | **** |  | 0,3072 | -0,02971 to 0,6440 | No | ns |
| DLD-1 vs. C33A | -0,72 | -1,293 to -0,1474 | Yes | ** |  | 0,4054 | 0,06852 to 0,7423 | Yes | * |
| HeLa vs. SKOV-3 | -0,33 | -0,9026 to 0,2426 | No | ns |  | 0,2612 | -0,07566 to 0,5981 | No | ns |
| HeLa vs. A-2780 | 0,59 | 0,01744 to 1,163 | Yes | * |  | 0,153 | -0,1838 to 0,4899 | No | ns |
| HeLa vs. MCF-7 | -1,04 | -1,613 to -0,4674 | Yes | *** |  | 0,09216 | -0,2447 to 0,4290 | No | ns |
| HeLa vs. MDA-MB-231 | -0,86 | -1,433 to -0,2874 | Yes | ** |  | 0,2531 | -0,08375 to 0,5900 | No | ns |
| HeLa vs. C33A | -0,33 | -0,9026 to 0,2426 | No | ns |  | 0,3514 | 0,01448 to 0,6882 | Yes | * |
| SKOV-3 vs. A-2780 | 0,92 | 0,3474 to 1,493 | Yes | *** |  | -0,1082 | -0,4451 to 0,2287 | No | ns |
| SKOV-3 vs. MCF-7 | -0,71 | -1,283 to -0,1374 | Yes | * |  | -0,169 | -0,5059 to 0,1678 | No | ns |
| SKOV-3 vs. MDA-MB-231 | -0,53 | -1,103 to 0,04256 | No | ns |  | -0,00808 | -0,3450 to 0,3288 | No | ns |
| SKOV-3 vs. C33A | 0 | -0,5726 to 0,5726 | No | ns |  | 0,09014 | -0,2467 to 0,4270 | No | ns |
| A-2780 vs. MCF-7 | -1,63 | -2,203 to -1,057 | Yes | **** |  | -0,06086 | -0,3977 to 0,2760 | No | ns |
| A-2780 vs. MDA-MB-231 | -1,45 | -2,023 to -0,8774 | Yes | **** |  | 0,1001 | -0,2368 to 0,4370 | No | ns |
| A-2780 vs. C33A | -0,92 | -1,493 to -0,3474 | Yes | *** |  | 0,1983 | -0,1385 to 0,5352 | No | ns |
| MCF-7 vs. MDA-MB-231 | 0,18 | -0,3926 to 0,7526 | No | ns |  | 0,161 | -0,1759 to 0,4978 | No | ns |
| MCF-7 vs. C33A | 0,71 | 0,1374 to 1,283 | Yes | * |  | 0,2592 | -0,07768 to 0,5961 | No | ns |
| MDA-MB-231 vs. C33A | 0,53 | -0,04256 to 1,103 | No | ns |  | 0,09823 | -0,2386 to 0,4351 | No | ns |

| **Tab3s** |  | CYP1B1 mRNA (RTPCR) | |  |  |  | CYP1B1 protein (Western blot) | | |
| --- | --- | --- | --- | --- | --- | --- | --- | --- | --- |
| Tukey's multiple comparisons test | Mean Diff, | 95% CI of diff, | Significant? | Summary |  | Mean Diff, | 95% CI of diff, | Significant? | Summary |
| DLD-1 vs. HeLa | -0,18 | -0,5072 to 0,1472 | No | ns |  | -1,137 | -1,530 to -0,7429 | Yes | **** |
| DLD-1 vs. C33A | 0,17 | -0,1572 to 0,4972 | No | ns |  | 0,3062 | -0,08757 to 0,6999 | No | ns |
| DLD-1 vs. A-2780 | 0,01 | -0,3172 to 0,3372 | No | ns |  | -0,5514 | -0,9452 to -0,1577 | Yes | ** |
| DLD-1 vs. MCF-7 | -0,08 | -0,4072 to 0,2472 | No | ns |  | 0,3214 | -0,07237 to 0,7151 | No | ns |
| DLD-1 vs. LOVO | 0,77 | 0,4428 to 1,097 | Yes | **** |  | 1,336 | 0,9419 to 1,729 | Yes | **** |
| DLD-1 vs. MDA-MB-231 | -0,53 | -0,8572 to -0,2028 | Yes | *** |  | -0,1319 | -0,5257 to 0,2618 | No | ns |
| DLD-1 vs. SKOV-3 | -0,8 | -1,127 to -0,4728 | Yes | **** |  | -0,1261 | -0,5199 to 0,2677 | No | ns |
| HeLa vs. C33A | 0,35 | 0,02277 to 0,6772 | Yes | * |  | 1,443 | 1,049 to 1,837 | Yes | **** |
| HeLa vs. A-2780 | 0,19 | -0,1372 to 0,5172 | No | ns |  | 0,5852 | 0,1914 to 0,9790 | Yes | ** |
| HeLa vs. MCF-7 | 0,1 | -0,2272 to 0,4272 | No | ns |  | 1,458 | 1,064 to 1,852 | Yes | **** |
| HeLa vs. LOVO | 0,95 | 0,6228 to 1,277 | Yes | **** |  | 2,472 | 2,079 to 2,866 | Yes | **** |
| HeLa vs. MDA-MB-231 | -0,35 | -0,6772 to -0,0227 | Yes | * |  | 1,005 | 0,6109 to 1,398 | Yes | **** |
| HeLa vs. SKOV-3 | -0,62 | -0,9472 to -0,2928 | Yes | *** |  | 1,011 | 0,6168 to 1,404 | Yes | **** |
| C33A vs. A-2780 | -0,16 | -0,4872 to 0,1672 | No | ns |  | -0,8576 | -1,251 to -0,4638 | Yes | **** |
| C33A vs. MCF-7 | -0,25 | -0,5772 to 0,07723 | No | ns |  | 0,0152 | -0,3786 to 0,4090 | No | ns |
| C33A vs. LOVO | 0,6 | 0,2728 to 0,9272 | Yes | *** |  | 1,029 | 0,6357 to 1,423 | Yes | **** |
| C33A vs. MDA-MB-231 | -0,7 | -1,027 to -0,3728 | Yes | **** |  | -0,4381 | -0,8319 to -0,04437 | Yes | * |
| C33A vs. SKOV-3 | -0,97 | -1,297 to -0,6428 | Yes | **** |  | -0,4323 | -0,8260 to -0,03853 | Yes | * |
| A-2780 vs. MCF-7 | -0,09 | -0,4172 to 0,2372 | No | ns |  | 0,8728 | 0,4790 to 1,267 | Yes | **** |
| A-2780 vs. LOVO | 0,76 | 0,4328 to 1,087 | Yes | **** |  | 1,887 | 1,493 to 2,281 | Yes | **** |
| A-2780 vs. MDA-MB-231 | -0,54 | -0,8672 to -0,2128 | Yes | *** |  | 0,4195 | 0,02571 to 0,8132 | Yes | * |
| A-2780 vs. SKOV-3 | -0,81 | -1,137 to -0,4828 | Yes | **** |  | 0,4253 | 0,03155 to 0,8191 | Yes | * |
| MCF-7 vs. LOVO | 0,85 | 0,5228 to 1,177 | Yes | **** |  | 1,014 | 0,6205 to 1,408 | Yes | **** |
| MCF-7 vs. MDA-MB-231 | -0,45 | -0,7772 to -0,1228 | Yes | ** |  | -0,4533 | -0,8471 to -0,05957 | Yes | * |
| MCF-7 vs. SKOV-3 | -0,72 | -1,047 to -0,3928 | Yes | **** |  | -0,4475 | -0,8412 to -0,05373 | Yes | * |
| LOVO vs. MDA-MB-231 | -1,3 | -1,627 to -0,9728 | Yes | **** |  | -1,468 | -1,861 to -1,074 | Yes | **** |
| LOVO vs. SKOV-3 | -1,57 | -1,897 to -1,243 | Yes | **** |  | -1,462 | -1,856 to -1,068 | Yes | **** |
| MDA-MB-231 vs. SKOV-3 | -0,27 | -0,5972 to 0,05723 | No | ns |  | 0,005836 | -0,3879 to 0,3996 | No | ns |

Fig. 1s. Plots IC_50_ values obtained for resveratrol and its analogue 3,3’,4,4’,5,5’-*trans*-hexahydroxystilbene (M12) in cytotoxicity study versus Western blot band intensities of CYP1A1, CYP1B1 and MnSOD. The panels show following relationships: A) Resveratrol versus MnSOD, B) M12 versus MnSOD, C) Resveratrol versus CYP1A1, D) M12 versus CYP1A1,  E) Resveratrol versus CYP1B1, F) M12 versus CYP1B1
